# Performance and Submaximal Adaptations to Additional Speed-Endurance Training Vs. Continuous Moderate-Intensity Aerobic Training in Male Endurance Athletes

**DOI:** 10.2478/hukin-2022-0060

**Published:** 2022-09-08

**Authors:** Vincenzo Rago, Peter Krustrup, Magni Mohr

**Affiliations:** 1 Faculty of Health Sciences and Sports, Universidade Europeia, Lisbon, Portugal; 2 Department of Sports Science and Clinical Biomechanics, SDU Sport and Health Sciences Cluster (SHSC), University of Southern Denmark, Odense, Denmark; 3 Centre of Health Science, Faculty of Health, University of the Faroe Islands, Tórshavn, Faroe Islands

**Keywords:** anaerobic training, maximal oxygen uptake, blood lactate, submaximal exercise, performance

## Abstract

We examined performance and submaximal adaptations to additional treadmill-based speed-endurance training (SET) vs. continuous moderate-intensity aerobic training (MIT) twice / week. Twenty-two male endurance athletes were tested before and after 10-week SET (6-12 × 30-s sprints separated by 3-min rest intervals) and MIT (2040 min continuous running at ~70% maximal oxygen uptake [V̇O_2max_]). The SET group attained greater acute heart rate (HR) and blood lactate responses than the MIT group (d = 0.86–0.91). The SET group improved performance in a time-to-exhaustion trial, V̇O_2max_, and lactate threshold (d = 0.50–0.73), whereas no training-induced changes were observed in the MIT group. Additionally, the SET group reduced oxygen uptake, mean HR and improved running economy (d = 0.53–0.86) during running at 10 and 12 km·h^-1^. Additional SET imposes greater physiological demands than MIT resulting in superior performance adaptations and reduced energy cost in endurance athletes.

## Introduction

Endurance training is mostly based on high volumes with most of the time exercising at submaximal intensities (e.g. continuous moderate-intensity aerobic training; MIT (Laursen, 2010)). As aerobic energy supply dominates the energy requirements of endurance athletes, most of the endurance training is generally aimed at increasing aerobic capacity (Laursen, 2010). Nonetheless, performance in endurance events (e.g., rowing, swimming, running and cycling) involves energy contribution from both aerobic and anaerobic sources (Laursen, 2010). However, it is unclear whether performance and cardiorespiratory adaptations are affected by additional endurance training.

Beyond MIT, endurance training is also prescribed using higher intensities, shorter exercise intervals and longer recovery periods compared to MIT (e.g., high-intensity training; (Buchheit and Laursen, 2013a, 2013b; Iaia and Bangsbo, 2010; MacInnis and Gibala, 2017)). By default, high-intensity training is based on a maximum of 30-min duration with multiple periods of near-maximal to maximal efforts, interspersed by longer recovery periods (Buchheit and Laursen, 2013a, 2013b). Particularly, speed-endurance training (SET; i.e., repeated bouts of all-out or based on 90–95% maximum sprinting effort lasting <40 s; work-to-rest ratio 1:5) has shown to promote comparable performance and physiological adaptations to that of conventional

MIT (Bangsbo, 2015; Iaia and Bangsbo, 2010). However, sparse research is available about the SET-induced effects on physiological and performance variables (Bangsbo, 2015; Hostrup and Bangsbo, 2017; Iaia and Bangsbo, 2010; Mohr and Krustrup, 2016).

Endurance performance primary relies on maximal oxygen uptake (VO_2max_), running economy (RE) as well as reduced heart rate (HR) responses during submaximal exercise (Bangsbo, 2015). VO_2max_ indicates the maximal amount of energy available through the oxidative process per unit of time during exhaustive activities, being a central component of endurance activities (Bassett and Howley, 2000), whereas RE represents the oxygen cost required to perform a specific activity at submaximal intensities and provides information about an athlete’s aerobic efficiency to perform such a given task (Barnes and Kilding, 2015). After a period of SET, several positive performance effects have been observed in runners (Bangsbo et al., 2009; Bickham et al., 2006; Skovgaard et al., 2018; Vorup et al., 2016) and soccer players (Fransson et al., 2018; Gunnarsson et al., 2012; Mohr and Krustrup, 2016; Nyberg et al., 2016). On the other hand, unclear effects have been observed on VO_2max_ (Bangsbo et al., 2009; Skovgaard et al., 2014), with improved endurance performance associated with a reduction in energy cost (EC) during submaximal exercise (e.g., RE; Bangsbo et al., 2009; Skovgaard et al., 2014, 2018), and various muscle mechanisms that may delay fatigue during intense exercise (Hostrup and Bangsbo, 2017; Skovgaard et al., 2014, 2018).

Beyond SET-induced performance adaptations, information regarding submaximal cardiorespiratory adaptations to SET in endurance athletes is limited to one study (Skovgaard et al., 2018). Thus, the purpose of the present study was to test the hypothesis that additional SET induces greater improvements in endurance performance, VO_2max_ and RE than MIT in elite male endurance athletes.

## Methods

### Participants

Twenty-four elite male endurance athletes were initially recruited for the study. However, there were two dropouts from the study (one from each group due to injuries), resulting in a final sample of 22 participants (Table 1) from different endurance sports (long-distance swimming, *n* = 4; long-distance running, *n* = 4; rowing, *n* = 6; and different team sports, *n* = 8). Participants had a weekly training volume of 6-10 sessions of approximate duration of 90-150 min per session. The inclusion was based on the following selection criteria: (a) national competitive standard with a central important endurance component (i.e., 800-1500 m freestyle swimming, 5-10 km distance running, 2000 m rowing, team handball or soccer); (b) injury-free during the last 3 months prior to the study; (c) VO_2max_ >55 ml·min^-1^·kg^-1^, (d) competitive background of minimum 3 years; (e) male aged 18-25 yrs. All athletes were part of the national team of the Faroe Islands in their respective sports. The study was approved by the local ethics committee of the Faroe Islands (*Vísindasisemisnevndin*) and conducted in accordance with the Declaration of Helsinki. After being informed verbally and formally about the experimental procedures and associated risks, all participants gave their written consent to take part in the study.

**Table 1:** Participants’ baseline characteristics (n = 22).

Group	Age (yrs)	Height (cm)	Body mass (kg)	VO_2max_ (ml·kg^-1^·min^-1^)	ITT (min)
SET	22.0 ± 1.8	184.9 ± 5.4	76.9 ± 5.4	60.82 ± 2.66	5.76 ± 0.42
MIT	21.9 ± 2.3	182.4 ± 7.9	73.0 ± 6.1	60.56 ± 3.10	5.77 ± 0.45

Data are presented as means ± SEM; SET, speed-endurance training; ITT, incremental treadmill test; MIT, moderate-intensity aerobic training; VO_2max_, maximal oxygen uptake.

### Measures

A longitudinal (pre- and post-training) design was applied. Participants were matched according to their sport modality and randomly assigned to either a SET group (n = 11) or a MIT group (n = 11). The randomization process first separated the participants in groups, ensuring an equal representation from each sport under random and blinded conditions.

Before and after the intervention, endurance performance and cardiorespiratory responses were assessed. Physiological measurements were performed on a motorized treadmill under standardized laboratory conditions (20°C; 40% relative humidity). On the testing day, participants arrived at the laboratory after consuming a light meal, refraining from caffeine and alcohol consumption during the 48 hrs before the experiment. To minimize diet-induced changes in physiological variables, participants were also required to record their individual food intake in the 48 hrs preceding the pre-training tests and to replicate their individual dietary pattern prior to post-training testing.

### Design and Procedures

#### Training intervention

SET and MIT were added to the participants’ regular training twice per week, always separated by two days. The intervention was performed in the morning (~7-8 a.m.) on a treadmill before the athletes’ regular practice. SET consisted of 6 to 12 reps of 30-s sprints at ~95% maximal effort (21-23 km·h^-1^) in each training session separated by 3-min rest intervals. The SET group performed 6 sprints during the 1^st^ to the 3^rd^ week, 9 sprints in the 4^th^ to the 6^th^ week, and 12 sprints in the 7^th^ to the 10^th^ week. The MIT group performed two weekly sessions of low-intensity continuous running at ~70% VO_2max_ (based on baseline testing; ~11.5-12.0 km·h^-1^) during 20 to 40 min. The MIT group ran for 20 min during the 1^st^ to the 3^rd^ week, 30 min during the 4^th^ to the 6^th^ week and 40 min during the 7^th^ to the 10^th^ week. Both SET and MIT protocols were preceded by a 15-min warm-up consisting of continuous running at 10 km·h^-1^. All sessions were carefully supervised by the athlete´s coaching and medical staff. Acute HR responses and capillary blood lactate were assessed during a random training session in week 5 to describe the exercise demands.

#### Incremental Treadmill Test

The treadmill slope was set at 1% to reproduce the energetic cost of outdoor running (Jones and Doust, 1996). The HR, rate of oxygen uptake (V^˙^O2), carbon dioxide expiration rate (V^˙^CO2) and ventilation (V_E_) were recorded over three submaximal 6-min running trials based on pre-established speeds (10, 12, and 14 km·h^-1^) separated by 2-min rest intervals (Barnes et al., 2014) and over an incremental treadmill test (ITT). Steady state condition and submaximal intensity during each stage were confirmed as maintenance of a respiratory exchange ratio (RER; calculated dividing VCO_2_ by VO_2_) of less than 1.0. After 10 min, participants started an ITT with a running speed of 16 km·h^-1^ with running speed increased by 1 km·h^-1^ each min until volitional exhaustion. The test was considered completed when participants were not able to withstand the physical effort imposed by the test, showing visual signs of volitional fatigue or wanted to stop the test because of discomfort, despite constant encouragement from the research team. Maximum effort was confirmed based on the presence of a plateau in oxygen uptake (maintenance of VO_2_ values; ± 2 ml·kg·min^-1^).

#### Physiological Measurements

V_O2_, VCO_2_ and V_E_ values were collected throughout the protocol using a breath-by-breath gas analysing system (Cosmed, Quark b2, Milan, Italy). The gas analyser was calibrated before each test with two gases of known oxygen and carbon dioxide concentrations as well as by the use of a 3-liter syringe for the tube flowmeter calibration (Porszasz et al., 1994). VO_2max_ was determined as the highest value achieved over a 20-s period. A plateau in VO_2_, despite an increased speed, and a RER >1.15 were adopted as criteria for VO_2max_ achievement. Additionally, the participants’ HR during the test was continuously recorded using a short-range telemetry device (Polar S610; Polar Electro Oy, Kempele, Finland) fitted around the chest and the data were collected at 5-s intervals, moreover, the mean HR during exercise (HR_ex_) was retained. The RE was calculated dividing pulmonary VO_2_ during the last minute of each running stage by running speed and body mass, and expressed as gross caloric unit cost (ml·kg^-1^·km^-1^) (Shaw et al., 2014). The VO_2_, VCO_2_, V_E_, HR_ex_ and RE values obtained during the last 3 min of each running stage were retained for analyses.

Upon completion of the ITT, peak blood lactate concentrations ([BLa¯]) were collected from the index finger tip using a hand-held portable analyser (Lactate Pro, Arkray, KDK, Japan) in 5 μL samples (Pettersen et al., 2014). The highest [BLa¯] value of two readings (immediately at exhaustion and 2 min after the completion of the ITT) was retained as the indicator of anaerobic glycolytic capacity (Green and Dawson, 1993).

### Statistical Analysis

The Shapiro-Wilk test revealed that all variables were normally distributed within each group and evaluation moment (*p* > 0.05). After assuming no significant between-group differences at baseline using an unpaired *t*-test (*p* > 0.05), a further *t*-test was employed to compare the acute training responses between groups in a random session on the fifth week. A two-way repeated-measures analysis of variance (ANOVA) was used on performance and physiological variables. The independent variables included one between-subjects factor (training intervention) with two levels (SET and MIT), and one within-subject factor (time) with two levels (pre- and post-intervention). To examine the influence of training intervention on the development of our dependent variables, we used these ANOVAs to test the null hypothesis of no difference in change over time between groups (time × group interaction). To interpret the magnitude of differences, effect sizes and associated 95% confidence intervals were classified as small, moderate and large (*d* = 0.2–0.5, 0.5–0.8, > 0.8; respectively) (Cohen, 1988).

Data were reported as mean ± standard deviation (SD) for all variables. Statistical significance was set at *p* ≤ 0.05. Analyses were performed using Statistical Package for Social Science software, version 25.0 (IBM, Armonk, NY).

## Results

The SET group largely attained higher peak and mean HR and peak [BLa¯] during a SET session than the MIT group (*d* = 0.86–0.91; *p* < 0.001; Table 2).

**Table 2:** Acute exercise responses to a random training session.

Variable	Speed-endurance training	Moderate-intensity training	*d* (95% CI)	*p*
HR_peak_ (%HR_max_)	96.23 ± 1.67 (93.37; 98.97)	82.56 ± 2.55 (77.00; 86.15)	0.91 (0.81; 0.97)	< 0.001
HR_ex_ (%HR_max_)	92.23 ± 1.64 (89.27; 95.00)	79.65 ± 2.24 (75.00; 82.56)	0.91 (0.81; 0.97)	< 0.001
Peak [BLa¯] (mmol·L^-1^)	11.43 ± 1.96 (8.20; 14.20)	3.55 ± 1.20 (1.90; 5.50)	0.86 (0.70; 0.94)	< 0.001

Descriptive statistics are mean ± standard deviation (range). BLa¯, blood lactate concentrations; CI, confidence intervals; HR_ex_, mean heart rate during exercise; HR_peak_, peak heart rate.

The SET group moderately improved VO_2max_, ITT results and [BLa¯] (*d* = 0.52 [0.17–0.88], 0.61 [0.25–0.96] and 0.50 [0.15–0.86], respectively; *p* < 0.05). However, no performance changes were observed after MIT (*p* > 0.05). A small time × group interaction was observed for the ITT and peak [BLa¯] (*d* = 0.47 [0.37–0.59] and 0.35 [0.14– 0.56], respectively; *p* < 0.05). A description of performance adaptations to SET and MIT is reported in Table 3.

**Table 3:** Performance adaptations to speed-endurance training (SET) and moderate-intensity training (MIT) in endurance athletes.

	SET (n = 11)		MIT (n = 11)		Time × Group interaction	

Variable	Pre	Post	Pre	Post	*d* (95% CI)	*p*
VO_2max_ (ml·kg^-1^·min^-1^)	60.82 ± 2.66	62.56 ± 3.42 ^M^	60.56 ± 3.10	61.10 ± 3.81	0.11 (-0.04; 0.26)	0.129
TTE trial (min)	5.76 ± 0.42	5.95 ± 0.46 ^M^	5.77 ± 0.45	5.71 ± 0.43	0.47 (0.37; 0.59)	< 0.001
Bla¯ (mmol·L^-1^)	11.04 ± 1.81	11.88 ± 1.88 ^M^	10.56 ± 1.88	10.13 ± 1.35	0.35 (0.14; 0.56)	0.004

Data are presented as means ± SEM; BLa¯, blood lactate concentrations, TTE, time to exhaustion; V̇O_2max_, maximal oxygen uptake. The superscript letters denote the magnitude of differences compared to “Pre” where ^S^ is small (d = 0.2–0.5), ^M^ is moderate (d = 0.5–0.8) and ^L^ is large (d > 0.8) effect size (p ≤ 0.05).

The SET group largely reduced VO_2_ and VCO_2_ when running at 10 and 12 km·h^-1^ (*d* = 0.81– 0.86; *p* < 0.05). On the other hand, the MIT group showed small-to-moderate increases in VO_2_ during exercise (*d* = 0.58 [0.23–0.94], 0.37 [0.03– 0.71] and 0.35 [0.01–0.68] at 10, 12 and 14 km·h^-1^, respectively; *p* < 0.05). Additionally, when running at 10 and 12 km·h^-1^, the SET group experienced small-to-moderate reductions in HR_ex_ (*d* = 0.76 [0.62–0.90] and 0.42 [0.07–0.77], respectively; *p* < 0.05) and improved RE (*d* = 0.60 [0.25–0.94] and 0.53 [0.18–0.89] respectively; *p* < 0.05). Contrarily, when running at 10 and 12 km·h^- 1^, the MIT group presented a slightly increased HR_ex_ (*d* = 0.40 [0.05–0.75] and 0.46 [0.11–0.82], respectively; *p* < 0.05) and impaired RE (*d* = 0.41 [0.07–0.76] and 0.29 [0.03–0.61], respectively; *p* < 0.05). Small to moderate time × groups interactions were observed for both VO_2_, VCO_2_, HR_ex_ and RE at 10 and 12 km·h^-1^ (*d* = 0.40–0.77; *p* < 0.05). A description of submaximal cardiorespiratory adaptations to SET and MIT is reported in Table 4.

**Table 4:** Submaximal cardiorespiratory adaptations to speed-endurance training (SET) and moderate-intensity training (MIT) in endurance athletes.

		SET (n = 11)		MIT (n = 11)		Time × Group interaction	

Variable	Intensity (km·h^-1^)	Pre	Post	Pre	Post	*d* (95% CI)	*p*
VO_2_	10	2.87 ± 0.25	2.77 ± 0.24 ^L^	2.57 ± 0.27	2.61 ± 0.26 ^M^	0.77 (0.59; 0.94)	< 0.001
(L·kg^- 1^·min^-1^)	12	3.38 ± 0.30	3.27 ± 0.27 ^L^	2.96 ± 0.35	3.00 ± 0.35 ^S^	0.67 (0.52; 0.83)	< 0.001
	14	3.86 ± 0.32	3.84 ± 0.33	3.53 ± 0.43	3.58 ± 0.44 ^S^	0.17 (-0.01; 0.34)	0.058

VCO_2_	10	2.51 ± 0.21	2.41 ± 0.19 ^L^	2.19 ± 0.24	2.21 ± 0.22	0.68 (0.53; 0.84)	< 0.001
(L^⸱^ min^-1^)	12	3.07 ± 0.26	2.95 ± 0.26 ^L^	2.65 ± 0.32	2.66 ± 0.30	0.56 (0.43; 0.68)	< 0.001
	14	3.67 ± 0.27	3.63 ± 0.31	3.33 ± 0.45	3.36 ± 0.43	0.18 (-0.01; 0.37)	0.051

V^E^	10	61.91 ± 10.66	63.18 ± 10.43	54.18 ± 6.63	54.91 ± 7.04	0.01 (-0.04; 0.06)	0.683
(L^⸱^ min^-1^)	12	76.09 ± 11.67	77.91 ± 11.39	69.27 ± 8.10	69.82 ± 8.44	0.04 (-0.05; 0.13)	0.390
	14	91.73 ± 10.89	93.00 ± 11.39	83.91 ± 8.85	85.82 ± 9.01	0.01 (-0.04; 0.06)	0.661

HR_ex_	10	73.94 ± 2.40	72.37 ± 2.00 ^M^	72.65 ± 2.07	73.89 ± 2.07 ^S^	0.56 (0.43; 0.69)	< 0.001
(%HR_max_)	12	82.12 ± 2.51	81.07 ± 2.85 ^S^	81.33 ± 3.32	82.49 ± 3.12 ^S^	0.44 (0.22; 0.66)	0.001
	14	88.4 ± 2.98	87.49 ± 2.89	89.88 ± 3.62	90.25 ± 3.45	0.13 (-0.03; 0.29)	0.094

RE	10	224.00 ± 12.84	218.16 ± 11.82 ^M^	211.31 ± 18.79	215.29 ± 18.24 ^S^	0.51 (0.40; 0.63)	< 0.001
(ml·kg^- 1^·min^-1^)	12	219.55 ± 13.97	214.74 ± 11.34 ^M^	202.84 ± 18.83	206.46 ± 18.75 ^S^	0.40 (0.18; 0.62)	0.002
	14	215.17 ± 12.29	216.47 ± 9.70	207.47 ± 20.58	210.94 ± 20.71	0.03 (-0.05; 0.12)	0.423

Data are presented as means ± SEM; HR_ex_, mean heart rate during exercise; HR_max_, maximum heart rate; RE, running economy; RER, respiratory exchange ratio; VCO_2_, carbon dioxide release; VO_2_, mean pulmonary oxygen uptake; V_E_, pulmonary ventilation rate. The superscript letters denote the magnitude of differences compared to “Pre” where ^S^ is small (d = 0.2–0.5), ^M^ is moderate (d = 0.5–0.8) and ^L^ is large (d > 0.8) effect size (p ≤ 0.05).

## Discussion

The present study provides further support to SET to improve performance in athletes. Specifically, additional speed endurance training based on 30-s sprints at ~95% maximal effort seems to produce greater submaximal cardiorespiratory adaptations compared to continuous MIT (~70% VO_2max_) in male elite endurance athletes, which are likely to be mediated by higher acute physiological responses during training.

SET imposed greater physiological demands than the MIT intervention. Specifically, both HR_peak_ and HR_ex_ were largely higher during SET than MIT, indicating greater cardiovascular stimulation. As HR_ex_ is closely related to VO_2_ during exercise when expressed as the percentage of individual HR_max_ (Achten and Jeukendrup, 2003) and to perception of effort (Marcora, 2009) during running, a higher cardiovascular and perceptual load might be expected when using SET compared to MIT. Additionally, the greater peak [BLa¯] during SET indicates a higher anaerobic contribution to the energy yield compared to MIT. This is confirmed by previous findings in active adults performing cycling-based SET based on six 20-s bouts of all-out cycling at 140% maximum power, compared to eight 60-s bouts at 85% maximum power and six 2-min bouts at 70% maximum power (Olney et al., 2018). Additionally, Mohr et al. (2007) found high muscle lactate concentrations after a SET session comparable to the present study. Our observed acute exercise responses are also supported by a recent work showing that SET may result in short-term (24 to 72 hrs) neuromuscular fatigue in soccer players (Tzatzakis et al., 2019). Since athletes in the present study trained 6-10 times per week in addition to the intervention protocol, SET sessions may have affected their performance during normal training.

SET induces superior performance improvements compared to MIT, which are in line with extensive research in long-distance runners (Bangsbo et al., 2009; Bickham et al., 2006; Skovgaard et al., 2018; Vorup et al., 2016) and team sport athletes (Fransson et al., 2018; Mohr and Krustrup, 2016; Nyberg et al., 2016; Purkhús et al., 2016). Our observed changes in VO_2max_ after the SET intervention contrast with previous studies showing unaltered VO_2max_ in runners performing 30-s sprints 3–4 times/week compared to continuous running training (~55 km/week) (Bangsbo et al., 2009) or heavy-resistance training (89-90% one-maximum repetition) (Skovgaard et al., 2014). One reason for the diverging results may be related to our participant group which included rowers, swimmers, and team sport athletes, who were unfamiliar with treadmill running, and its associated biomechanical stimulus, compared to long-distance runners. Additionally, athletes assigned to the SET group may have had an improved glycolytic capacity as denoted by increased peak blood lactate after the ITT protocol.

Most submaximal cardiorespiratory adaptations to SET or MIT were observed at running intensities of 10 and 12 km·h^-1^. The lack of positive adaptations when running at 14 km·h^-1^ could be attributed to the fact that athletes reached the anaerobic threshold before this speed, as indicated by the appearance of an oxygen uptake slow component at this speed. In the present study, the SET group experienced large reductions in VO_2_ and VCO_2_ when running at 10 and 12 km·h^-1^. This is supported by findings in endurance runners performing 8–12 reps of 30-s sprints separated by 3-min rest intervals for 4 weeks (Iaia et al., 2009; Skovgaard et al., 2018). On the other hand, irrespective of the running intensity, the MIT group surprisingly increased VO_2_ during exercise. Potentially, additional MIT may have negatively affected normal training due to acute fatigue associated with training volume (Bangsbo et al., 2009). Alternatively, this type of training may not have been powerful enough to cause significant adaptations in muscular variables associated with locomotor efficiency. Furthermore, meaningful reductions in HR_ex_ and EC (improved RE) when running at 10 and 12 km·h^-1^ after the SET intervention, might be explained by concurrent reductions in VO_2_ during exercise. The main cause of the improved running seems to be related to muscular factors such as improved biomechanical factors (Pizzuto et al., 2019) and upregulated mitochondrial efficiency (Buchheit and Laursen, 2013a), as no difference was observed in pulmonary ventilation and only a minor part of lower VO_2_ after SET may relate to the reduced cardiac work (Kitamura et al., 1972). Our improved RE after SET is supported by the recent study of Skovgaard et al. (2018) in elite runners. However, our decreased submaximal HR_ex_ after SET is in contrast to studies in endurance athletes despite of reduced VO_2_ in these studies (Iaia et al., 2009; Skovgaard et al., 2018). In this context, our participants were elite athletes at the national level in a small country, and thus, their training status may have been at a sub-elite level compared to the international elite level in bigger countries. On the other hand, surprisingly the MIT group experienced small increases in HR_ex_ and EC (impaired RE), which could be partially explained by the additionally imposed demands of MIT.

It is important to denote some limitations inherent to this work. First, despite the equal number of sport representatives distributed in the two intervention groups, athletes represented different sporting modalities. Second, the two training interventions were compared with the absence of a classical control group. Third, it was not possible to monitor the training responses during the entire period. Fourth, we adopted arbitrary intensity zones to assess submaximal cardiorespiratory responses.

Taken together, our findings suggest that SET imposes greater physiological demands compared to continuous MIT in elite male endurance athletes. These demands seem to result in superior adaptations for endurance performance with a concurrently reduced EC during submaximal running. Endurance coaches can incorporate both treadmill-based SET and MIT to their regular in-season training programs to promote further gains in endurance performance and cardiorespiratory fitness. Nonetheless, SET might be preferred to MIT when the physiological target is to increase the anaerobic contribution to exercise. Caution should be paid to the exercise mode (e.g., running, swimming, rowing) which could affect the imposed sport-specific demands.

In conclusion, additional speed endurance training based on short intense bouts (30-s sprints at ~95% maximal effort) produces greater acute responses compared to continuous moderate-intensity aerobic training (~70% VO_2max_) during a 10-wk competitive period in male elite endurance athletes, resulting in superior performance and submaximal cardiorespiratory adaptations.
